# RNA-Seq Analysis Reveals Genes Underlying Different Disease Responses to Porcine Circovirus Type 2 in Pigs

**DOI:** 10.1371/journal.pone.0155502

**Published:** 2016-05-12

**Authors:** Yanping Li, Hao Liu, Pengfei Wang, Liyuan Wang, Yi Sun, Gen Liu, Ping Zhang, Li Kang, Shijin Jiang, Yunliang Jiang

**Affiliations:** Shandong Provincial Key Laboratory of Animal Biotechnology and Disease Control and Prevention, College of Animal Science and Veterinary Medicine, Shandong Agricultural University, Taian, China; Hospital Universitario LA FE, SPAIN

## Abstract

Porcine circovirus type 2 (PCV2), an economically important pathogen, causes postweaning multisystemic wasting syndrome (PMWS) and other syndrome diseases collectively known as porcine circovirus-associated disease (PCVAD). Previous studies revealed breed-dependent differences in porcine susceptibility to PCV2; however, the genetic mechanism underlying different resistance to PCV2 infection remains largely unknown. In this study, we found that Yorkshire × Landrace (YL) pigs exhibited serious clinical features typifying PCV2 disease, while the Laiwu (a Chinese indigenous pig breed, LW) pigs showed little clinical symptoms of the disease during PCV2 infection. At 35 days post infection (dpi), the PCV2 DNA copy in YL pigs was significantly higher than that in LW pigs (*P* < 0.05). The serum level of IL-4, IL-6, IL-8, IL-12 and TGF-β1 in LW pigs and TNF-α in YL pigs increased significantly at the early infected stages, respectively; while that of IL-10 and IFN-γ in YL pigs was greatly increased at 35 dpi. RNA-seq analysis revealed that, at 35 dpi, 83 genes were up-regulated and 86 genes were down-regulated in the lung tissues of LW pigs, while in YL pigs, the numbers were 187 and 18, respectively. In LW pigs, the differentially expressed genes (DEGs) were mainly involved in complement and coagulation cascades, metabolism of xenobiotics by cytochrome P450, RIG-I-like receptor signaling and B cell receptor signaling pathways. Four up-regulated genes (*TFPI*, *SERPNC1*, *SERPNA1*, and *SERPNA5*) that are enriched in complement and coagulation cascades pathway were identified in the PCV2-infected LW pigs, among which the mRNA expression of *SERPNA1*, as well as three genes including TGF-β1, TGF-β2 and VEGF that are regulated by *SERPNA1* was significantly increased (*P* < 0.05). We speculate that higher expression of *SERPNA1* may effectively suppress excessive inflammation reaction and reduce the pathological degree of lung tissue in PCV2-infected pigs. Collectively, our findings indicate that the susceptibility to PCV2 infection depends on a genetic difference between LW and YL pigs, and *SERPNA1* likely plays an important role in the resistance of LW pigs to PCV2 infection.

## Introduction

Porcine circovirus (PCV) is a small, non-enveloped virus of the *Circoviridae* family, with a small, circular, single-stranded DNA molecule of approximately 1.7 kb in size [1–4]. Genetic analysis identified two PCV genotypes: PCV1 and PCV2 [5, 6]. Studies have shown that PCV2 is the major genotype associated with PMWS [7–9]. The clinical signs of PCV2 infection include wasting, increased mortality, respiratory difficulties, enteritis, and reproductive disorders, collectively named porcine circovirus disease (PCVD) or porcine circovirus associated disease (PCVAD) [10]. Pigs infected with PCV2 also show lymphocyte depletion, interstitial pneumonia and hepatitis [11–14].

PCV2 virus alters the cytokine responses and leads to cytokine imbalance through targeting the lymphoid tissues and immune cells [15–17]. The cytokines and chemokines released by inflammatory cells were essential factors in resisting pathogen infection; however, the imbalance between anti-inflammatory and pro-inflammatory cytokines may contribute to the development of undesired tissue lesions [18]. Studies revealed that IL-10, IL-1 and TNF-α in serum were up-regulated, while IL-2 and IL-4 were down-regulated in PCV2-infected pigs with PCVAD [14, 19]. Up-regulation of IL-10 expression in the thymus associated with thymic depletion and atrophy [20] and elevated IL-10 and serum PCV2 level [21–23] were also reported in PCV2-infected pigs. Besides, PCV2 infection induces strong IL-1β and IL-8 responses in the peripheral blood mononuclear cells [24] and higher level of TNF-α, G-CSF, MCP-1, and IL-8 in alveolar macrophage [25, 26].

Genetic background is reported to influence clinical performances of pigs caused by PCV2 infection [27]. For example, Landrace pigs are more susceptible than Duroc and Large White pigs to PCV2 infection [28]; Landrace pigs infected with PCV2 had significantly severer lymphoid lesions than Pietrain pigs, while Large White × Duroc crossbred pigs were more susceptible to PCV2 infection than purebred Pietrain pigs [29]. Moreover, PCV2 replication patterns in pulmonary alveolar macrophages displayed clear differences among macrophages from different conventional crossbred pigs [30].

The objective of this study was to determine the effect of PCV2 infection on performance and disease traits of two genetically distinct pig breeds known as Laiwu (a Chinese indigenous pig breed, LW) and Yorkshire × Landrace (YL) pigs, and to explore the genetic mechanism underlying different responses to PCV2 infection between the two pig breeds. We found that PCV2 infection triggered an excessive immune and inflammatory response in the YL pigs, and this contributed to severe tissue damage, particularly lung damage but mild symptoms in LW pigs, and the higher expression of *SERPINA1* in lung tissue was identified as an important mechanism preventing lung damage induced by PCV2 infection in LW pigs.

## Materials and Methods

### Animals and PCV2 infection

Thirty 6-week-old (on average) weaned purebred LW pigs and crossbred YL pigs were randomly sampled from Laiwu Pig Preservation Center (Laiwu, China) and a pig farm in the Changqing District of Jinan city (Jinan, China), respectively. All pigs were both antigen and antibody seronegative for PCV1, PCV2, porcine reproductive and respiratory syndrome virus (PRRSV) and porcine parvovirus (PPV) as determined by ELISA assays (Shandong Center for Disease Control and Prevention, Jinan, China). The pigs were randomly assigned to four groups, namely, LW-i (n = 10), LW-u (n = 5), YL-i (n = 10) and YL-u (n = 5), and were raised in isolation rooms of the same experimental base which could control the spread of the disease effectively and eliminate the effect of environment on the results. PCV2-SD was isolated from pigs that were infected with PMWS in Shandong province and used in this experiment. The virus genome of PCV2-SD is 1767bp in size and the homology of nucleotide sequence between PCV2-SD and reference sequence (NC_005148.1) from GenBank is more than 96%. Pigs from LW-i and YL-i groups were infected intramuscularly with 3mL (10^3.8^ tissue culture infective dose 50/mL) of the PCV2-SD strain to ensure that each pig received the same dose at the time of infection. The uninfected groups (LW-u and YL-u) were treated similarly with an identical volume of phosphate buffered saline (PBS). The raise, inoculation and daily management on pigs were approved by the Institutional Animal Care and Use Ethics Committee of Shandong Agricultural University and performed in accordance with the “Guidelines for Experimental Animals” of the Ministry of Science and Technology (Beijing, China).

### Clinical evaluation and sample collection

During the experimental period, clinical signs were monitored daily and the pigs were weighed until 35 dpi. Anticoagulant-treated blood and untreated blood samples were collected separately by venipuncture at 0, 4, 7, 10, 14, 21, 28 and 35 dpi. Blood samples were collected for measuring the PCV2 virus copy number and cytokines. At 35 dpi, all pigs were sacrificed through electric stun and bled from heart in 15s, which were complied with the Standard of Animal Management of Shandong Agricultural University, and tissue samples including lung, tonsil, thymus, ileum, kidney, spleen, and liver were collected and frozen in liquid nitrogen. Pathological lesions in organs were recorded for each pig. The remaining portions of the above organs were preserved and fixed by immersion in 10% neutral-buffered formalin for subsequent histological examination.

### PCV2 DNA quantification

The DNA of the serum samples in PCV2-infected pigs at 7, 10, 14, 21, 28 and 35 dpi was extracted using a TIANamp Virus DNA /RNA Mini Kit (Tiangen, China) and used for quantifying the copy number of PCV2 genomic DNA by real-time PCR. Primers (F: 5′-TATCAAGCGAACCACAGTC-3′ and R: 5′-ATGGCGG-GAGGAGTAGTT-3′) were designed according to the published ORF2 sequence of PCV2 (EU780074). A 276 bp conserved region of the ORF2 gene of PCV2 was amplified by PCR and cloned into a pMD-18T vector (TaKaRa, Dalian, China). The resultant pMD-18T-276 plasmid was used as a standard DNA template to optimize the assay conditions. The PCV2 genomic DNA copy number in the pigs was analyzed by real-time quantitative PCR with the following conditions: 95°C for 30 s, 95°C for 5s, 55°C for 30 s and 72°C for 20 s for 30 cycles. The baseline adjustment method of the MX3000p software (Stratagene, La Jolla, CA, USA) was used to determine the Ct value for each reaction. The copy number of the sample was measured by a linear formula that is established according to the standard curve using the 10-fold serial dilutions of the standard plasmid. All samples were amplified in triplicate.

### Cytokine analysis by protein array

Blood samples collected at 0, 4, 7, 14, 21, 28 and 35 dpi of pigs were centrifuged at 2000 × g for 10 min, and the supernatant was isolated. Cytokine concentrations were measured by a microarray panel specific for swine serum (Quantibody Porcine Cytokine Array, RayBiotech, Inc.).The array format (#QAP-CYT-1-2 and #QAP-CYT-1-4) contains quadruplicate and duplicate antibody spots for cytokines GM-CSF/CSF2, IL-1β, IL-6, IL-10, TGF-β1, IFN-γ, IL-4, IL-8, IL-12p40 and TNF-α. Microarrays were scanned by GenePix4000B (Sunnyvale, CA, USA) at an excitation of 532 nm and cytokine level was quantified using Quantibody® Q-Analyzer software (http://www.raybiotech.com/).

### RNA-seq

Total RNA was extracted from the lung tissues of four PCV2-infected pigs and four uninfected pigs of each breed, through TRIzol® Reagent (Invitrogen Life Technologies, Carlsbad, CA, USA) according to the manufacturer’s instructions. The extracted RNA was treated with DNase (Promega, Madison, WI, USA) to remove potential genomic DNA contamination, according to the manufacturer’s protocol. Equal amounts of RNA from each of the four samples were mixed to build a library and, altogether, four libraries including LW-u, LW-i, YL-u and YL-i were constructed. A single 100 ng mRNA aliquot was reserved for evaluation on the Agilent 2100 Bioanalyzer (Agilent Technologies, Palo Alto, CA, USA). The mRNA was broken into short fragments, and the first-strand cDNA synthesis was performed using random hexamer primers and reverse transcriptase (Invitrogen). Second-strand cDNA was synthesized using RNase H (Invitrogen), DNA polymerase I (New England Biolabs, MA, USA), dNTPs and buffer. RNA-seq library construction and sequencing for each sample were performed according to the protocols for the Illumina HiSeq2000 to generate 10M paired-end reads. Quality-controlled reads were mapped to the *Sus scrofa* genome (Sscrofa9.65) from NCBI using SOAPaligner/SOAP2 and were counted using HTseq (v0.5.3p3). The reads per kilobase per million (RPKM) for each gene was calculated based on the length of the gene, and the read counts mapped to it.

DEGs and their corresponding *P*-values were determined according to the methods described by Audic and Claverie [31]. The false discovery rate (FDR) was used to assess the *P*-value in multiple tests. Fold change (log_2_Ratio) was estimated according to the normalized gene expression level in each sample. The FDR and absolute value of log2Ratio were set to ≤0.001 and ≥1 as the threshold to judge significance differences in gene expression, respectively.

### Gene ontology (GO) and KEGG

First, all the DEGs were mapped to GO terms in the database (http://www.geneontology.org/), and gene numbers for every term were calculated. Next, a hypergeometric test was used to find significantly enriched GO terms in the DEGs compared with the genome background. The DEGs were then filtered according to their biological functions, and functional classification was applied to them. GO covers three domains: cellular component, molecular function and biological process. With non-redundant annotation, the Blast2GO program (version 2.3.5) was used to obtain GO annotation of the DEGs. After obtaining the GO annotation for the DEGs, WEGO software (http://wego.genomics.org.cn/cgi-bin/wego/index.pl) was used to perform GO functional classification of the DEGs to facilitate understanding of the distribution of gene functions in the species from the macro level in the GO database (http://www.geneontology.org/). KEGG pathway analysis of all the DEGs was performed using Cytoscape software (version 2.6.2) (http://www.cytoscape.org/) with the ClueGO plugin (http://www.ici.upmc.fr/cluego/cluegoDownload.shtml). The Q value of ≤ 0.05 in a pathway was defined as a significant enrichment of the pathway in the DEGs. Pathway enrichment analysis identifies significantly enriched metabolic pathways or signal transduction pathways in DEGs compared with the whole genome background.

### Real-time quantitative reverse transcriptase PCR (qRT-PCR)

The RNA samples used for the qRT-PCR assay were the same as those used for the RNA-seq. PrimeScript RT reagent Kit with gDNA Eraser (TaKaRa) was used for reverse transcription of RNA samples. The mRNA level of four DEGs (*TFPI*, *SERPNC1*, *SERPNA1* and *SERPNA5*), as well as *TGF-β1*, *TGF-β2*, *VEGF*, *IL-10*, *IL-1R*, *TLR2*, *TLR4*, *IL-6*, and *TNF-α* was measured by qRT-PCR with primers designed according to their gene sequences (Table 1). The *GAPDH* gene was selected as the internal control [32]. qRT-PCR was conducted on an MX3000p instrument (Stratagene, La Jolla, CA, USA) in a 15 μL volume containing 7.5 μL of 2 × SYBR Premix Ex Taq II (TaKaRa), 0.3 μL of 50 × Rox Reference Dye II, 0.3 μL of each forward and reverse primer (10 μM) and 2.0 μL of cDNA at a dilution of 1:4 according to the following program: 95°C for 30 s, 95°C for 5 s, annealing temperature (Table 1) for 20 s and 72°C for 20 s for 40 cycles. A 2-fold dilution series of cDNA was included in each run to determine the PCR efficiency by constructing a relative standard curve using SYBR×Premix Ex Taq (TaKaRa). A non-template negative control was included for each qRT-PCR reaction to check for primer-dimers. The baseline adjustment method of the MX3000 software (Stratagene, La Jolla, CA, USA) was used to determine the Ct of each reaction. The amplification efficiencies were close to 100%, using the 2^-△△ct^ method for calculating the relative gene expression levels of a sample. All samples were amplified in triplicate.

**Table 1:** Primer sequence used in this study for qRT-PCR in pigs.

Primer	Primer sequences (5'→3')	Product size (bp)	Annealing temperature (°C)
TFPI	F: GGAAAGGTATCTGTGAGGTGA	183	56.7
	R: CCAGACTGACCAAAATTAT		56.4
SERPINC1	F: GCCACCATCTTCTATCAGCA	214	58.8
	R: AGTCGGCAGTTCAGTTTGG		58.9
SERPINA1	F: CCTTCTCCATCAACTTCAGG	195	57.3
	R: TCCTCCTCTGTGGTTTGCT		58.4
SERPINA5	F: ACAAGCTTCAACCACAAAATCAC	129	60
	R: CTTGCAGGAGAGGTTTCGGT		50
TGF-β1	F: GTGGCTGTCCTTTGATGT	118	55
	R: CGTGGAGTGTGTTATCTTTG		55.8
TGF-β2	F: TTACAACACCCTCTGGCTC	144	57.6
	R: GCATTCTTCTCCATCGCT		55
VEGF	F: ATCTTCAAGCCGTCCTGT	197	55
	R: CGCTCTATCTTTCTTTGGTC		55.8
IL-10	F: CTGCATCCACTTCCCAACCA	218	59.8
	R: CCATCACTCTCTGCCTTCGG		61.9
IL-1R	F: CAGACCTGAGAAAGAACAG	184	55.4
	R: GTAGAACTTGGTGACCTTG		55.4
TNF-α	F: AACCTCAGATAAGCCCGT	167	55
	R: AGGACCTGGGAGTAGATGA		57.6
TLR2	F: GAGTTTAAAGACGGTGTGCTGC	257	60.1
	R: GGTCACTGCTGCCCACATAG		61.9
TLR4	F: CTCCAGCTTTCCAGAACTGC	151	59.8
	R: GTCCAGAAAAGGCTCCCAGG		61.9
IL-6	F: ACCAGGAACGAAAGAGAG	118	55
	R: CAGTAGCCATCACCAGAA		55
GAPDH	F: ACTCACATCTTCTACCTTTGATGCT	100	58.6
	R: TGTTGCTGTAGCCAAATTCA		53.7

### Western blotting

The SERPINA1 protein level in lung tissues was measured by Western blotting. Protein samples were extracted using Cell lysis buffer for Western and IP (Beyotime, Beijing, China). Thirty microgram of the protein samples was used as protein loading. Samples were transferred to polyvinylidene fluoride membrane (Solarbio, Beijing, China), which were then blocked for 2 h in Western Blocking Buffer (Beyotime) at 37°C. Membranes were incubated overnight at 4°C with the rabbit polyclonal antibody against SERPINA1 which was diluted 500 times (ABBIOTEC, Ste A, San Diego, USA) or a monoclonal antibody against GAPDH which was diluted 1000 times (Beyotime). Membranes were washed in PBST, and then incubated with HRP-labeled Goat Anti-Mouse IgG which were diluted 1000 times (Beyotime) for 1 h. Next, the proteins on the membranes that reacted with the antibodies were visualized using a DAB Horseradish Peroxidase Color Development Kit (Beyotime). Signals were captured through film coloration and measured using ImageJ software (http://imagej.nih.gov/ij/).

### Statistical analysis

The mean values shown in the figures and used in the statistical analyses represented at least three independent trials. Differences between each group were evaluated by ANOVA, followed by Duncan’s multiple-range test using the General Linear Model procedure of SAS (SAS version 8.2; Cary, NC, USA). Results with *P* values of < 0.05 were considered statistically significant when testing for differences among groups.

## Results

### Clinical and pathological outcomes for the PCV2-infected pigs

**Fig 1:**
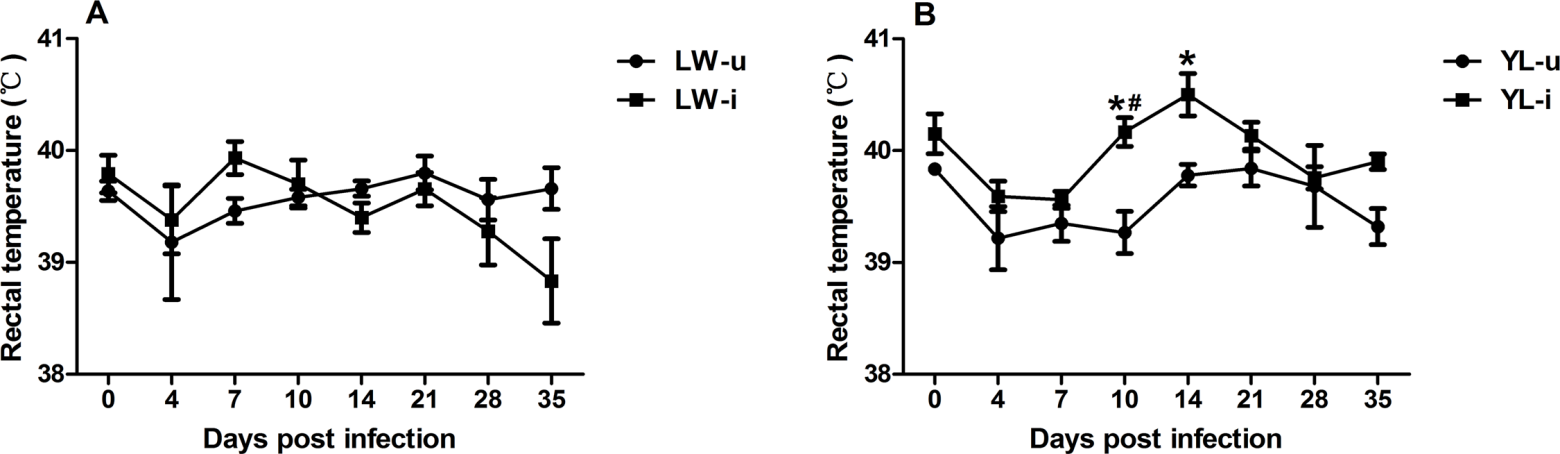
Changes in rectal temperature after PCV2 infection. The rectal temperature of LW-u pigs (A), LW-i pigs (A), YL-u pigs (B) and YL-i pigs (B) were collected at different dpi. The rectal temperature of YL-i pigs exceeded 40°C from 10 to 21dpi. * *P* < 0.05 versus YL-u group at same dpi, # *P* < 0.05 versus YL-i group at 7dpi.

**Fig 2:**
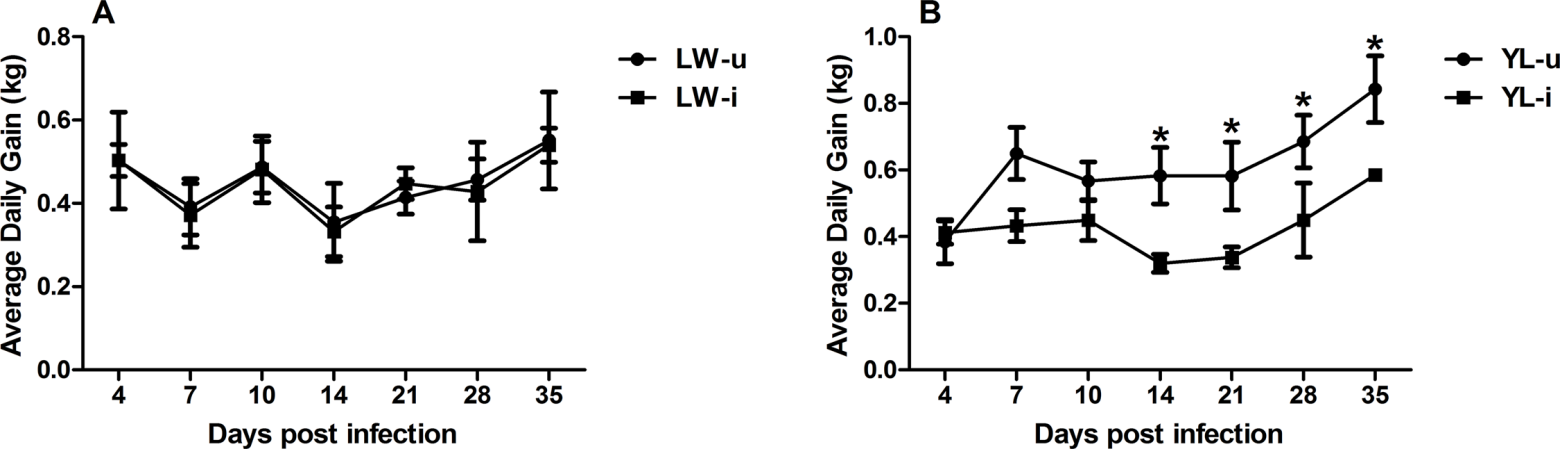
Changes in average daily gain after PCV2 infection. The average daily gain of LW-u pigs (A), LW-i pigs (A), YL-u pigs (B) and YL-i pigs (B) were collected at different dpi. The average daily gain of YL-i pigs decreased from 14 dpi compared with YL-u pigs. * *P* < 0.05 versus YL-i group at the same dpi.

**Fig 3:**
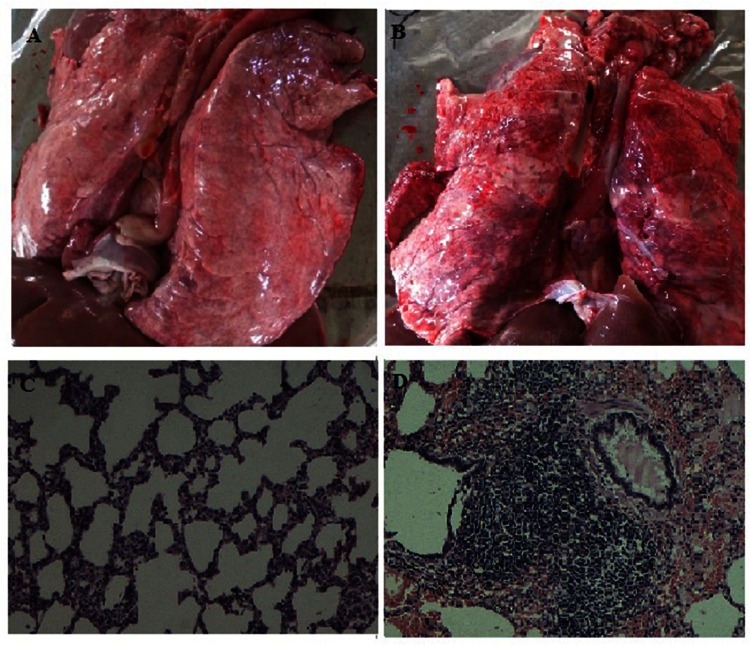
Pathological changes in lung tissue after PCV2 infection. The necropsy (A) and tissue slice (C) results of LW-i pigs showed normal signs. The necropsy (B) and tissue slice (D) results of YL-i pigs showed serious clinical signs: congestion, bleeding, interstitial pneumonia and lymphocyte infiltration in the bronchus determined by hematoxylin and eosin staining (× 200).

Sixty percent (6/10) of the PCV2-infected YL pigs exhibited the typical clinical signs of PMWS, such as reduced appetite, breathlessness, coughing, diarrhea, decline of growth rate, occasional sneezing, and the irregular, red macules and papules on the back and hind limbs from approximately 14 dpi onward; while the LW pigs showed slightly clinical signs: occasionally anorexia, coughing and pyrexia. The PCV2-infected YL pigs had a persistent high rectal temperature (> 40°C) from 10 dpi to 21 dpi (Fig 1B); however, the LW pigs showed normal temperature during the infection (Fig 1A). During PCV2 infection, the trend of weight gain in PCV2-infected LW pigs were consistent with uninfected LW pigs (Fig 2A), while the PCV2-infected YL pigs had significantly lower average daily gain of body weight from 14 dpi to 35 dpi compared with uninfected pigs (*P* < 0.05) (Fig 2B). The lung tissue of PCV2-infected YL pigs showed serious lesions: interstitial broadening of the lung lobules, serious hyperemia and hemorrhage, and a large amount of lymphocyte infiltration in the bronchus (Fig 3B and 3D). However, these pathological signs were not observed in the PCV2-infected LW pigs (Fig 3A and 3C).

### PCV2 DNA copies in serum samples

**Fig 4:**
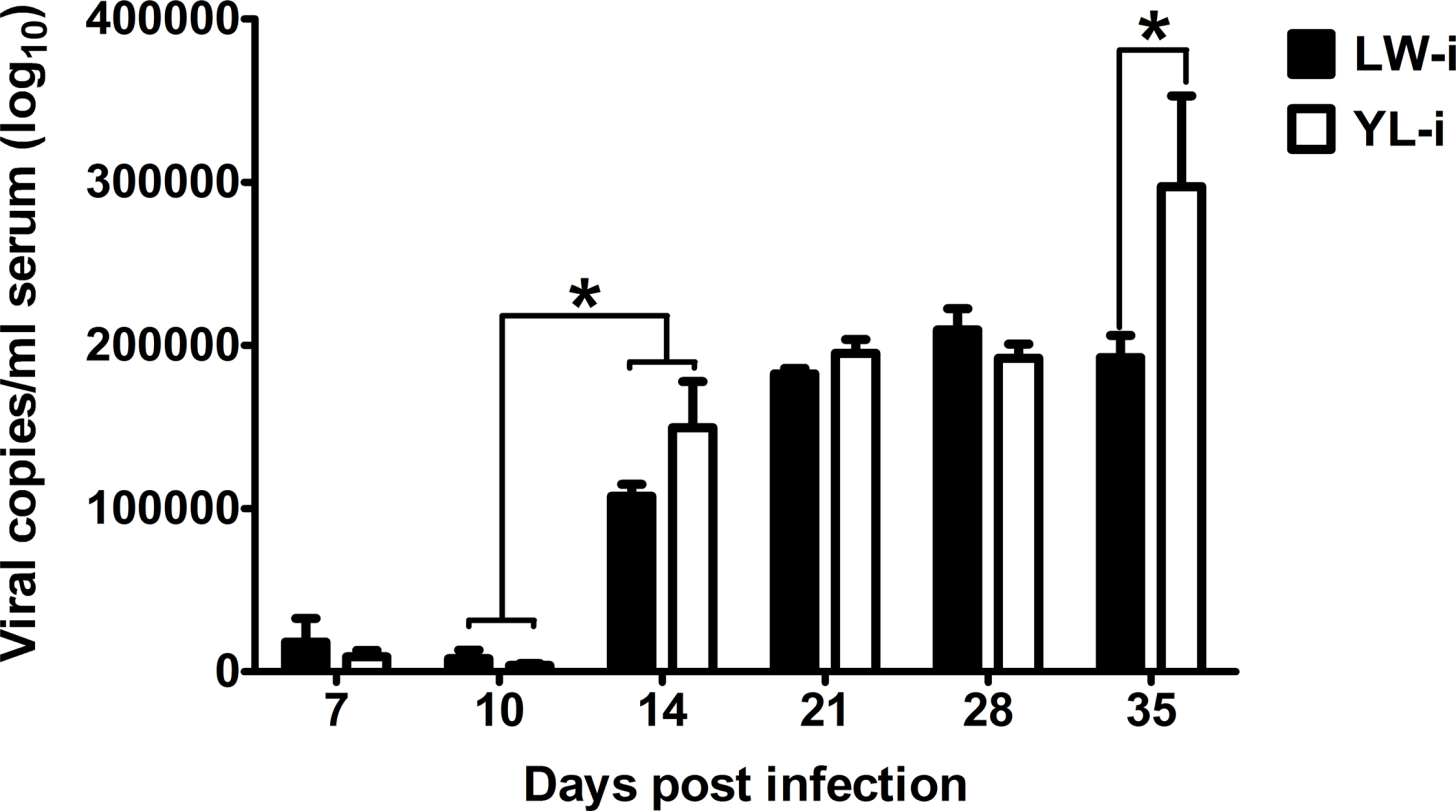
Changes in genomic copies of PCV2 in serum after PCV2 infection. The genomic copies of PCV2 in serum were determined through absolute quantitation PCR. Data were presented as the log10 transformed group means. The PCV2 copies of YL-i pigs were significantly higher than that of LW-i pigs at 35 dpi. * *P* < 0.05.

The copy number of PCV2 DNA in porcine serum was calculated with the obtained linear formula (Ct = -3.18× Log (copy number) +7.64) through the standard curve. The mean copy number of PCV2 DNA in the serum samples of pigs significantly increased in both PCV2-infected LW and YL pigs at 14dpi (*P* < 0.05), but not significantly different between PCV2-infected LW and YL pigs at 7, 10, 14, 21, and 28 dpi (*P* > 0.05). However, the copies of PCV2 DNA in the PCV2-infected YL pigs were significantly higher than that in the PCV2-infected LW pigs at 35 dpi (*P* < 0.05) (Fig 4).

### Cytokine level in porcine serum after PCV2 infection

**Fig 5:**
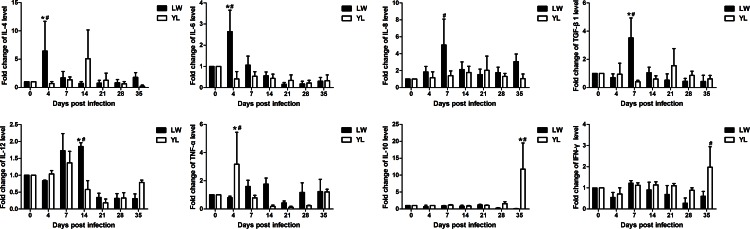
Changes in cytokine levels after PCV2 infection. The fold changes of cytokine levels in serum after PCV2 infection were calculated between LW-i and YL-i pigs. The levels of IL-4 (A), IL-6 (B), IL-8 (C), TGF-β1 (D) and IL-12 (E) in LW pigs were significantly increased at the early stage of PCV2 infection. The level of TNF-α (F) in YL pigs was significantly increased at 4dpi. And the levels of IL-10 (G) and IFN-γ (H) in YL pigs were significantly increased at 35 dpi. * *P* < 0.05 versus YL (or LW) group at same dpi, # *P* < 0.05 versus 0 dpi.

The fold change of cytokines in porcine serum of PCV2-infected pigs *vs* uninfected ones was compared between LW and YL pigs (Fig 5). A significant increase of IL-4 and Il-6 at 4 dpi, of IL-8 and TGF-β1 at 7 dpi, and of IL-12 at 14 dpi, was found in PCV2-infected LW pigs (*P* < 0.05), and their levels were significant higher than in YL pigs except IL-8 (*P* < 0.05). For TNF-α, however, the fold change was significantly increased in PCV2-infected YL pigs *vs* uninfected ones (*P* < 0.05) and was significantly higher than in LW pigs (*P* < 0.05). The fold change of IL-10 and IFN-γ at 35 dpi was significantly higher in PCV2-infected YL pigs *vs* uninfected ones, respectively (*P* < 0.05), and that of IL-10 was significantly higher than LW pigs (*P* < 0.05). No significant changes were found in the protein level of GM-CSF/CSF2 and IL-1β in PCV2-infected pigs (data not shown).

### Analysis of digital gene expression libraries

Four cDNA sequencing libraries of lung tissues collected from the PCV2-infected and uninfected LW and YL pigs were constructed through using the High-seq 2000 sequencing platform at BGI-Shenzhen, China. Clean reads with an average of ten million per library were obtained by filtering the following: adaptor sequences, sequences where the percentage of unknown bases (N) was greater than 10%, and low quality reads. After removing the adaptors and filtering, more than 99% reads were qualified as clean reads (S1 Fig). When mapping the clean reads to pig genome, 51% and 50.16% were perfectly matched, and 61.2% and 60.41% were uniquely matched in YL-i and YL-u pigs, respectively (Table 2); while 46.81% and 46.72% were perfectly matched, and 59.97% and 59.94% were uniquely matched in LW-i and LW-u pigs, respectively (Table 2). Sequence saturation analysis indicated that the growth curve for the genes detected flattened when the number of reads reached ten million, which indicates that the number of genes detected tends to saturate (S2 Fig). Analysis of the distribution of reads for the reference genes showed that the reads in every position were evenly distributed (S3 Fig). The gene coverage distributions were similar in the four RNA-seq libraries. As shown in S4 Fig, 27% (YL-u group), 26% (YL-i group), 31% (LW-u group) and 34% (LW-i group) of the reference genes had 90–100% coverage, respectively. The sequencing reads were submitted to the NCBI GEO (Gene Expression Omnibus) database under accession number [SRP064897].

**Table 2:** Statistics of sequencing reads aligned to genome for different pigs.

Sample ID	Total Reads	Total Base Pairs	Total Mapped Reads	Perfect Match	< = 2bp Mismatch	Unique Match	Multi-position Match	Total Unmapped Reads
YL-i	10,075,686(100.00%)	493,708,614(100.00%)	7,275,069(72.20%)	5,138,723(51.00%)	2,136,346(21.20%)	6,166,041(61.20%)	1,109,028(11.01%)	2,800,617(27.80%)
YL-u	10,400,431(100.00%)	509,621,119(100.00%)	7,444,251(71.58%)	5,217,055(50.16%)	2,227,196(21.41%)	6,283,208(60.41%)	1,161,043(11.16%)	2,956,180(28.42%)
LW-u	11,155,036(100.00%)	546,596,764(100.00%)	7,872,915(70.58%)	5,211,430(46.72%)	2,661,485(23.86%)	6,686,163(59.94%)	1,186,752(10.64%)	3,282,121(29.42%)
LW-i	10,349,002(100.00%)	507,101,098(100.00%)	7,321,365(70.74%)	4,844,038(46.81%)	2,477,327(23.94%)	6,206,805(59.97%)	1,114,560(10.77%)	3,027,637(29.26%)

### DEGs in the lung tissue of PCV2-infected pigs

**Fig 6:**
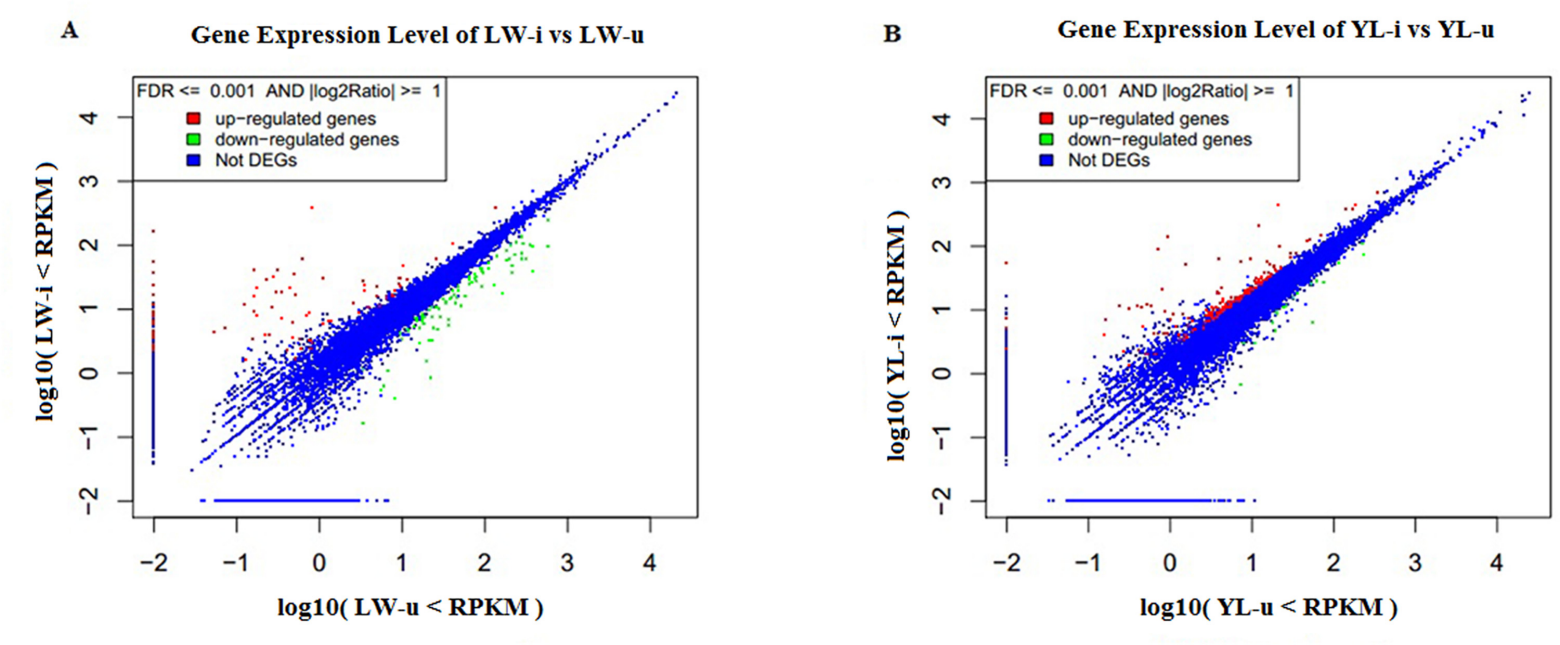
**Comparison of gene expression levels in the lung tissues between the LW-i and LW-u (A) and YL-i and YL-u (B) libraries.** The X and Y-axes show the mRNA expression levels in the two samples. Up-regulated and down-regulated genes were shown in red and green, respectively. Blue dots represent genes with similar expression levels.

Gene expression levels were evaluated by counting the number of the RPKM (S1 Table). In the four libraries, there were 12,988 (36.84%), 13,101 (37.16%), 12,913 (36.63%) and 12,971 (36.80%) known reference genes expressed in the lung tissues of LW-u, LW-i, YL-u and YL-i pigs, respectively. One hundred and sixty-nine DEGs were found in the LW-i *vs* LW-u pigs, with 83 genes being up-regulated and 86 genes down-regulated (S2 Table and Fig 6A). Two hundred and five DEGs were found in the YL-i *vs* YL-u pigs, with 187 genes being up-regulated and 18 genes down-regulated (S3 Table and Fig 6B). Among these DEGs, five were up-regulated and four were down-regulated in both breeds, while 152 genes (77, up-regulated and 75, down-regulated) were only differentially expressed in the LW-i *vs* LW-u pigs and 188 genes (175, up-regulated and 13, down-regulated) were only differentially expressed in the YL-i *vs* YL-u pigs (S4 Table). Subsequently, the UniProt (http://www.uniprot.org/) protein names of these expressed genes were identified by querying the bioDBnet (http://biodbnet.abcc.ncifcrf.gov/db/db2db.php#biod) database.

### GO functional enrichment analysis of DEGs

Using the Blast2GO software (version 2.3.5) in the GO database (http://www.geneontology.org/), the physiological functions and biological processes of the DEGs were classified into the following categories: molecular function, cellular component, and biological process. Both 169 DEGs (LW-i *vs* LW-u group) and 205 DEGs (YL-i *vs* YL-u group) were categorized into 47 functional groups, including 23 biological processes, 13 cellular components and 11 molecular function annotations (S5 Fig). Notably, some DEGs were significantly enriched in the defense response, response to cytokine stimulus, cytokine-mediated signaling pathway, response to stimulus, acute inflammatory response, negative regulation of blood coagulation and regulation of coagulation in the LW-i *vs* LW-u group (*P* < 0.05) (Table 3).

**Table 3:** Information of the significantly enriched GO terms.

Groups	GO ID	GO term	*P*-value	Gene counts
YL-i *vs* YL-u	GO:0031012	extracellular matrix	2.58E-05	15
	GO:0005576	extracellular region	0.00453	22
	GO:0044421	Extracellular region part	0.00471	21
	GO:0004673	Protein histidine kinase activity	0.02433	9
	GO:0016775	Phosphotransferase activity, nitrogenous group as acceptor	0.02433	3
LW-i *vs* LW-u	GO:0005576	extracellular region	1.13E-21	44
	GO:0044421	extracellular region part	5.14E-20	41
	GO:0061135	endopeptidase regulator activity	1.64E-12	17
	GO:0004866	endopeptidase inhibitor activity	2.14E-12	16
	GO:0030414	peptidase inhibitor activity	8.72E-12	16
	GO:0006952	defense response	5.34E-08	24
	GO:0034097	response to cytokine stimulus	1.91E-06	14
	GO:0019221	cytokine-mediated signaling pathway	3.59E-05	10
	GO:0050896	response to stimulus	9.77E-05	83
	GO:0002526	acute inflammatory response	0.0021	7
	GO:0030195	negative regulation of blood coagulation	0.01365	4
	GO:0050818	regulation of coagulation	0.01752	5

### DEGs pathway analysis

To further explore the biological pathways that were involved in the DEGs, we performed KEGG analysis of DEGs. With a P-value of < 0.05 and a FDR value of < 0.05 as criteria, ten pathways including the “Complement and coagulation cascades”, “Metabolism of xenobiotics by cytochrome P450”, “Drug metabolism—cytochrome P450” were significantly enriched in LW-i *vs* LW-u group (Table 4) and seven pathways including the “Protein digestion and absorption”, “Focal adhesion” and “ECM-receptor interaction” were significantly enriched in the YL-i *vs* YL-u group (*P* < 0.05) (Table 4).

**Table 4:** Significantly enriched pathways for DEGs in porcine lung tissues.

Groups	Pathway	DEGs with pathway annotation (%)	All genes with pathway annotation (%)	*P*-value
YL-i *vs* YL-u	Protein digestion and absorption	11 (6.75%)	148 (1.05%)	1.21E-06
	Focal adhesion	15 (9.2%)	391 (2.79%)	5.12E-05
	ECM-receptor interaction	10 (6.13%)	198 (1.41%)	0.00010599
	Fc epsilon RI signaling pathway	5 (3.07%)	113 (0.81%)	0.01022222
	Salmonella infection	7 (4.29%)	236 (1.68%)	0.02038835
	Amoebiasis	6 (3.68%)	190 (1.35%)	0.02359847
	Phagosome	7 (4.29%)	285 (2.03%)	0.04884937
LW-i *vs* LW-u	Complement and coagulation cascades	18 (12.59%)	150 (1.07%)	1.21E-14
	Metabolism of xenobiotics by cytochrome P450	9 (6.29%)	68 (0.48%)	2.73E-08
	Drug metabolism—cytochrome P450	8 (5.59%)	59 (0.42%)	1.37E-07
	Retinol metabolism	7 (4.9%)	67 (0.48%)	5.15E-06
	Arachidonic acid metabolism	8 (5.59%)	95 (0.68%)	5.51E-06
	Influenza A	12 (8.39%)	271 (1.93%)	2.13E-05
	Linoleic acid metabolism	5 (3.5%)	39 (0.28%)	4.47E-05
	Herpes simplex infection	11 (7.69%)	254 (1.81%)	5.81E-05
	Hepatitis C	8 (5.59%)	164 (1.17%)	0.000274659
	RIG-I-like receptor signaling pathway	5 (3.5%)	89 (0.63%)	0.002144184

### Validation of the DEGs involved in complement and coagulation cascades pathway

**Fig 7:**
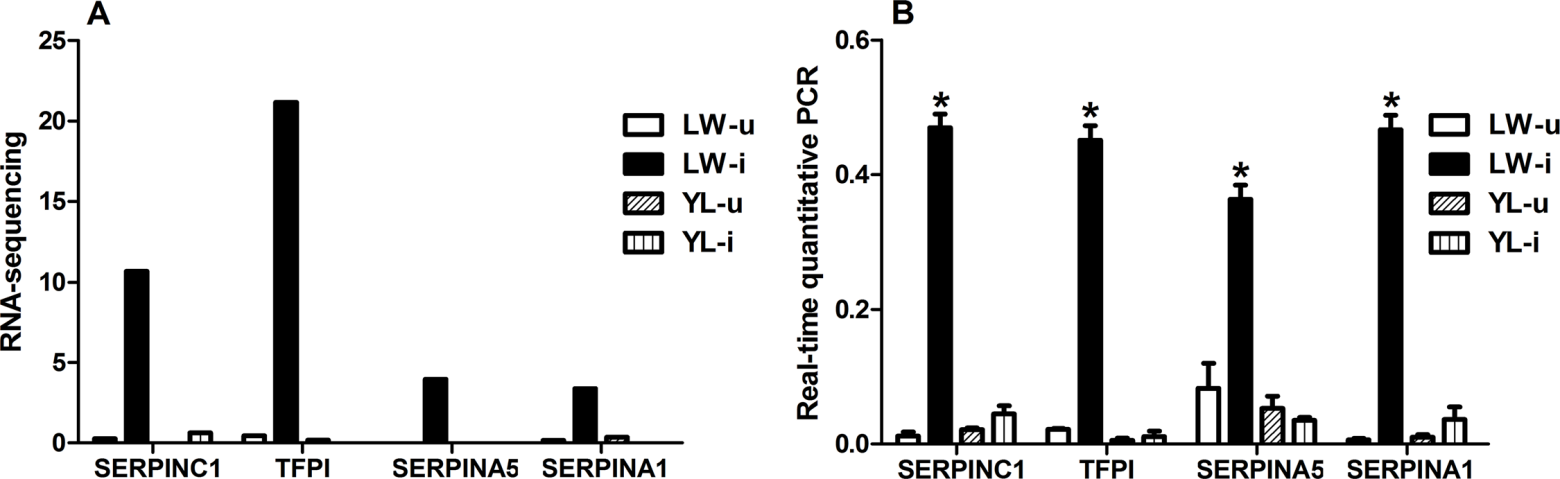
Changes in expression of the DEGs mRNA after PCV2 infection. The mRNA expression of the four DEGs was detected through qPCR. The results of qPCR were similar to the results of RNA-seq. The *GAPDH* gene was used as reference gene. * *P* < 0.05.

Four DEGs (*TFPI*, *SERPINC1*, *SERPINA1*, *and SERPINA5*) which were involved in complement and coagulation cascades pathway and up-regulated in LW-i *vs* LW-u group were validated by qRT-PCR. The fold change in the mRNA expression levels of the four genes as determined by qRT-PCR was consistent with the RNA-seq results (Fig 7). The correlations between the gene expression values obtained with RNA-seq and qRT-PCR were significant for the four genes (*P* < 0.01) (Table 5).

**Table 5:** qRT-PCR validation of RNA-seq results.

Gene	RNA-seq		qPCR		correlation(r)	*P* value (H0:Rh0 = 0)
	log2 Ratio(LW-i *vs* LW-u)	*P* value	log2 Ratio(LW-i *vs* LW-u)	*P* value		
*TFPI*	5.574	4.89E-38	4.365	0.027	0.99979	0.0002
*SERPINC1*	5.324	2.04E-21	5.267	0.02	0.9992	0.0008
*SERPINA5*	8.629	2.16E-08	2.135	0.0168	0.99203	0.008
*SERPINA1*	6.922	1.17E-34	6.173	0.0267	0.99047	0.0095

### Effect of PCV2 on SERPINA1 protein expression

**Fig 8:**
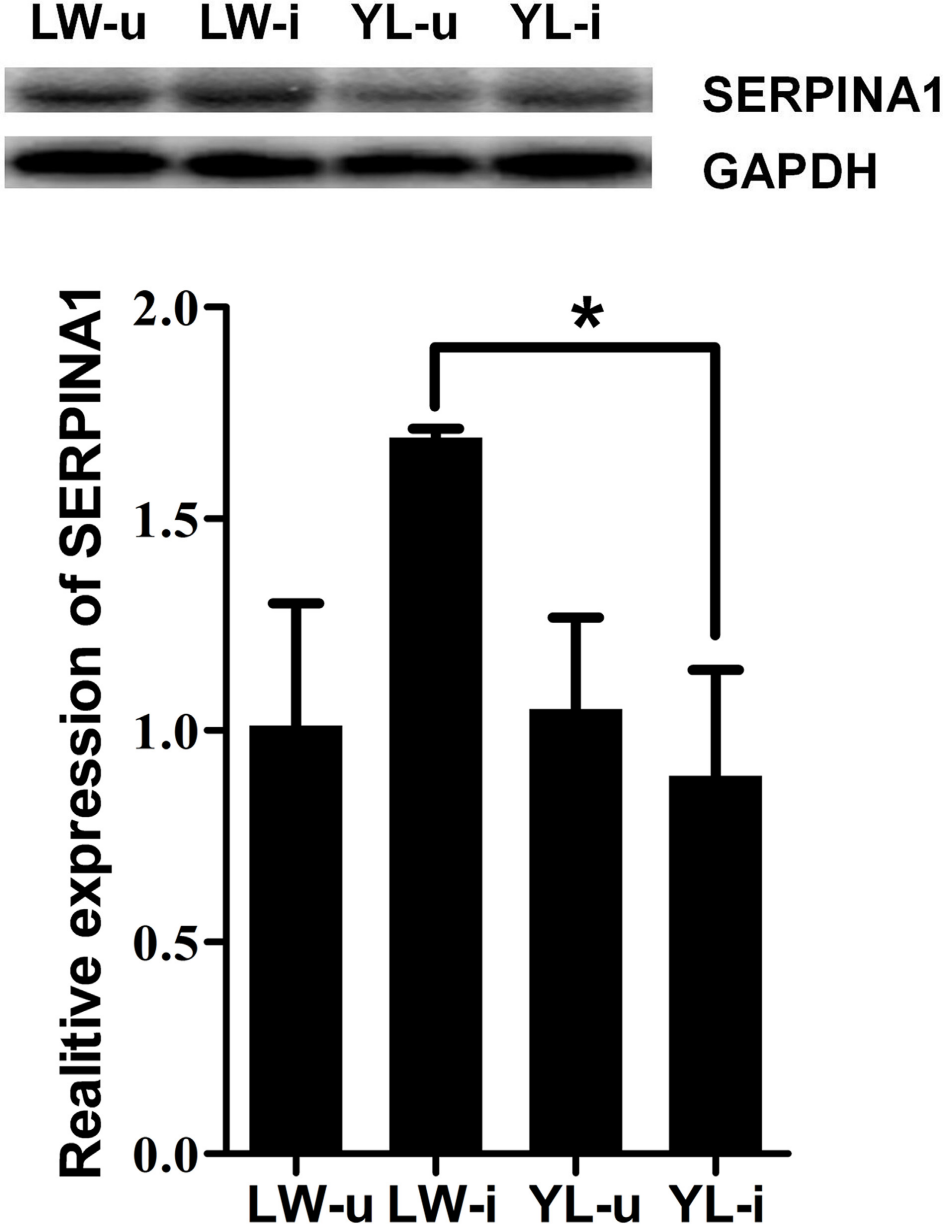
Changes in SERPINA1 protein expression after PCV2 infection. Western blotting was used to measure SERPINA1 protein expression in each group of lung tissue samples. The SERPINA1 level in LW-i was significantly higher than that in YL-i. GAPDH expression was used as the positive control. * *P* < 0.05.

To further investigate the effect of PCV2 infection on SERPINA1 protein expression, we examined the levels of SERPINA1 protein in the lung tissues by Western blotting. Compared with the *SERPINA1* mRNA level, the protein level in the lungs showed a similar trend. Although no significant difference was found in the SERPINA1 protein levels between the uninfected LW and PCV2-infected LW pigs, the SERPINA1 protein level in the lung tissue of PCV2-infected LW pigs was significantly higher than that of PCV2-infected YL pigs (*P* < 0.05) (Fig 8).

### Alterations in the mRNA expression of SERPINA1 regulated genes after PCV2 infection

**Fig 9:**
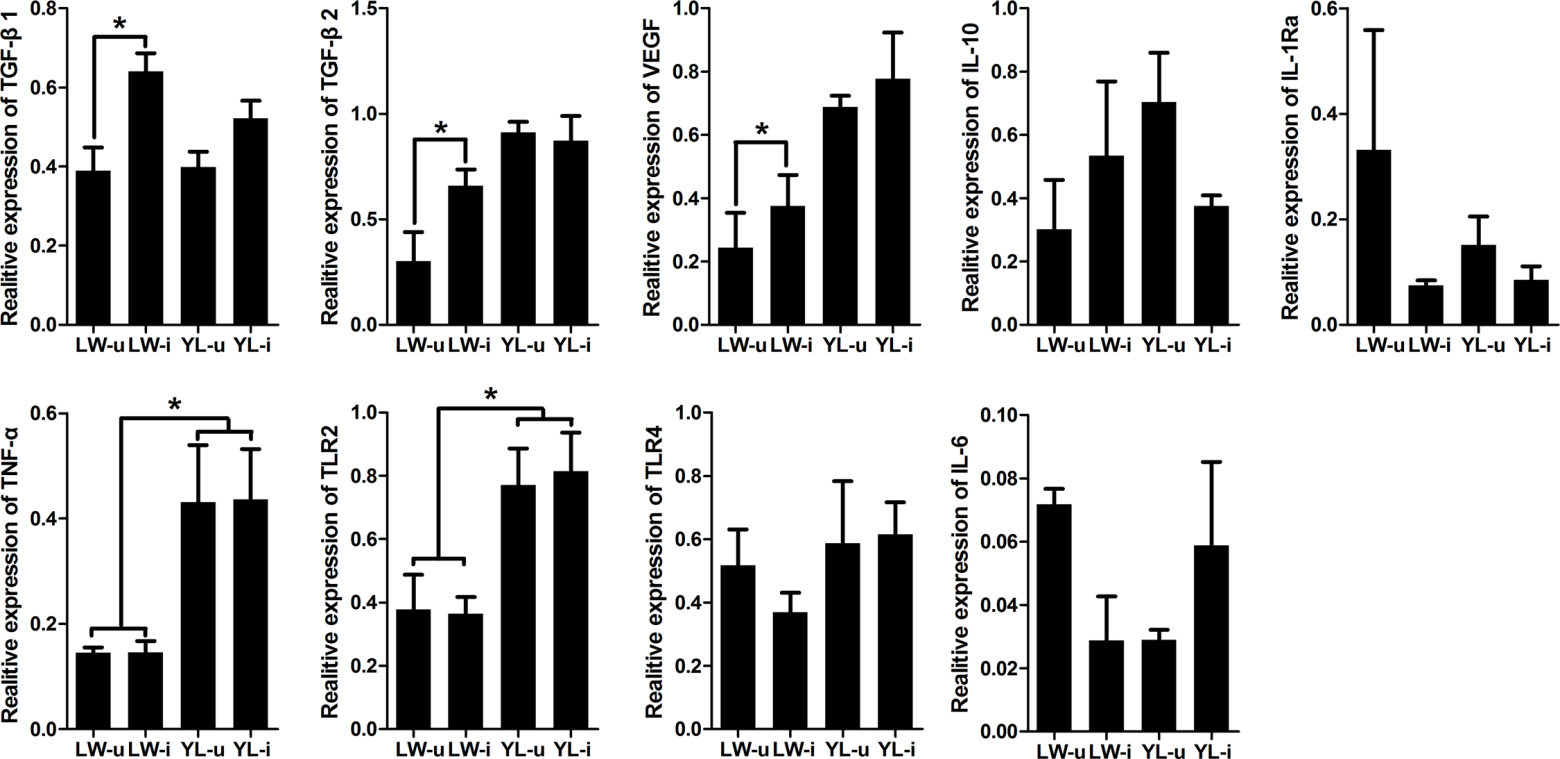
Changes in mRNA expression of SERPINA1-regulated genes after PCV2 infection. Real-time quantitative RT-PCR was used to assess the mRNA expression levels of *TGF-β1/2*, *VEGF*, *IL-10*, *IL-1Ra*, *TNF-α*, *TLR2*, *TLR4* and *IL-6* in the lung tissues of the four groups. Increased expression of *TGF-β1*, *TGF-β2* and *VEGF* mRNA was seen in LW pigs after PCV2 infection. *TNF-α*, and *TLR2* mRNA expression in YL-i was higher than that in LW-i. There was no significant difference in *IL-10*, *IL-1Ra*, *TLR4* and *IL-6* mRNA expression. * *P* < 0.05.

We further checked the expression of SERPINA1 regulated genes in porcine lung tissues and found that, *TGF-β1*, *TGF-β2* and *VEGF* mRNA levels in the PCV2-infected LW pigs were significantly higher than those of the uninfected LW pigs (*P* < 0.05). The mRNA expression levels of *TNF-α* and *TLR2* in YL pigs were significantly higher than those of LW pigs (*P* < 0.05), but no difference was found within both LW and YL pigs, suggesting that they are not affected by PCV2 infection. As for *IL-10*, *IL-1Ra*, *TLR4* and *IL-6*, no significant differences were detected among the four groups (*P* > 0.05) (Fig 9).

## Discussion

Respiratory distress is one of the main features of PMWS in some PCV2-infected pigs, and interstitial pneumonia and bronchiolitis with mononuclear infiltration have been observed in the lungs of such pigs [33, 34]. Meanwhile, PCV2 is also a potential cause of porcine dermatitis and nephritis syndrome (PDNS), which is characterized by coalescing red-to-purple skin lesions, glomerular and interstitial nephritis, and vasculitis [12, 35]. In this study, clinically different response to PCV2 infection was observed between LW and YL pigs, while PCV2-infected YL pigs showed typical clinical signs of PMWS, PCV2-infected LW pigs showed no such symptoms, which is consistent with previous report that different genetic background affects the resistance to PCV2 infection [28, 29]. The lymphoid depletion and histiocytic replacement in lymphoid tissue were not detected in both LW and YL pigs after PCV2 infection. This is likely due to that PCV2 infection alone does not produce the full spectrum of clinical PCVAD at 35 dpi.

As for the replication of PCV2 virus, we found that the PCV2 copy number significantly increased in both PCV2-infected LW and YL pigs at 14 dpi. However, at 35 dpi, it was significantly higher in PCV2-infected YL pigs than in PCV2-infected LW pigs (Fig 4). The results imply that more PCV2 viruses are the causes of the typical clinical symptoms observed in YL pigs after PCV2 infection, and the replication of PCV2 virus is more efficiently restricted in LW pigs.

The complex host-virus interaction and the subsequent immune response generated are critical to understanding PCV2-related disease [22, 36–39]. PCV2 primarily interacts with host cellular factors to modulate host immune function, which leads to cytokine imbalance, immunosuppression, and disease [16, 40]. For example, PCV2 infection induces TNF-α [14, 40] and IL-8 production in PBMCs [20, 24], and PMWS-affected pigs have elevated IL-10 levels [21, 22]. In this study, we found a breed-dependent difference in the expression of selected cytokines at the protein level. The elevation of pro-inflammatory cytokines (IL-6, IL-8 and IL-12) and anti-inflammatory cytokines (IL-4, TGF-β1) at the early stages of PCV2 infection in the serum of LW pigs suggest that LW pigs produce more pro-inflammatory cytokines after PCV2 infection to activate the immune system against PCV2 infection. At the same time, the elevation of anti-inflammatory cytokines in PCV2-infected LW pigs inhibits the excessive inflammatory reaction to maintain body homeostasis. Whereas in YL pigs, only TNF-α was significantly increased at the early stage of PCV2 infection, and the protein levels of IL-10 and IFN-γ in PCV2-infected YL pigs increased till at 35 dpi. These results reflect that YL pigs lack sensibility to PCV2 infection, and lead to the imbalance of body homeostasis and severe symptoms. Although IL-10 and IFN-γ level increased at 35 dpi, it might be too late to restore the health of the body in PCV2-infected YL pigs. Together, it is suggested that LW pigs exhibit more effective responses to PCV2 infection than YL pigs, which assisted LW pigs to resist PCV2 infection and sustain a relative stable homeostasis.

Due to PCV2-infected LW and YL pigs exhibited differences in their clinical appearance (especially in lung lesion) and cytokine levels, we further used RNA-seq high-throughput sequencing approach to examine mRNA expression profiles in the lung tissues of the four groups at 35 dpi. Four DEGs (*TFPI*, *SERPINC1*, *SERPINA1*, and *SERPINA5*) enriched significantly in the complement and coagulation cascade pathways were identified. Among these genes, SERPINA1 was up-regulated significantly in the PCV2-infected LW pigs compared with the uninfected pigs, while no marked changes were observed in the PCV2-infected YL pigs. SERPINA1 (serpin peptidase inhibitor, clade A, member 1), also called AAT, inhibits the activity of neutrophil enzymes like elastase, which could promote inflammatory responses, destroy tissue integrity [41], and disrupt neutrophil migration by binding to IL-8 [42]. The increase in burden (which might happen in pulmonary infections) or the quantitative deficiency of an inhibitor of neutrophil elastase can lead to an elastase/anti-elastase imbalance that accelerates lung tissue breakdown [41]. We speculate that the lower expression of SERPINA1 is a partia cause of the pathological signs including large areas of hyperemia, hemorrhage, and lymphocyte and neutrophil infiltration in the lung tissues of YL pigs infected with PCV2. The rise in SERPINA1 levels during the acute-phase response to PCV2-infection in the LW pigs highlights its importance as an angiogenesis and wound-healing modulator, and agrees with its antiviral and bacterial burden-reducing activities as previously reported [43].

Moreover, SERPINA1 can inhibit the production of IL-6 [44] and TNF-α [45] and down-regulates the synthesis of TLR2 and TLR4 by monocytes and neutrophils [46]. It also induces the production of VEGF [47] and TGF-β to promote wound healing and inhibit lymphocyte and endothelial cell adhesion [41]. Consistently, we found that, along with the increase of SERPINA1, the expression levels *TGF-β1*, *TGF-β2* and *VEGF* mRNA also significantly increased in the lung tissues of PCV2-infected LW pigs (Fig 9). The expression dynamics of *IL-6* and *TLR4* shows opposite trend to SERPINA1.We speculate that the increased expression of *SERPINA1* mRNA in the lung tissue of the PCV2-infected LW pigs results in a high level of SERPINA1 protein, thereby stimulating the expression of *TGF-β1*, *TGF-β2* and *VEGF*.

## Conclusions

In this study, we found that LW pigs showed mild clinical signs and had a lower PCV2 copy number (at 35 dpi) than YL pigs after infection with PCV2. Meanwhile, the levels of IL-4, IL-6, IL-8, TGF-β1 and IL-12 in LW pigs increased at the early stage of PCV2 infection; while IL-10 and IFN-γ increased in PCV2-infected YL pigs at 35 dpi. Four lung tissue damage-associated DEGs enriched in complement and coagulation cascades were up-regulated in infected LW pigs compared with their uninfected counterparts. The higher SERPNA1 expression level in the PCV2-infected LW pigs may effectively suppress the inflammation reaction and reduce the pathological changes in the lung tissue of the PCV2-infected LW pigs.

## Supporting Information

S1 Fig Composition of raw reads in the four groups. (TIF)

S2 Fig Percentage of genes mapped by clean reads in four groups. (TIF)

S3 Fig Distribution of reads for the reference genes. (TIF)

S4 Fig Distribution of gene’s coverage in four groups. (TIF)

S5 Fig GO functional enrichment analysis of differentially expressed genes. (TIF)

S1 Table Expression statistics for reference genes in the porcine transcriptome. (XLS)

S2 Table Differentially expressed genes in LW-i *vs* LW-u group. (XLS)

S3 Table Differentially expressed genes in YL-i *vs* YL-u group. (XLS)

S4 Table The comparison of differentially expressed genes between the LW-i *vs* LW-u group and YL-i *vs* YL-u group. (XLS)
