# Supplementary figures and images for: RNA-Seq Analysis Reveals Genes Underlying Different Disease Responses to Porcine Circovirus Type 2 in Pigs
Source: PLoS One. 2016 May 12;11(5):e0155502. doi: 10.1371/journal.pone.0155502 (PMC4865221; doi:10.1371/journal.pone.0155502)

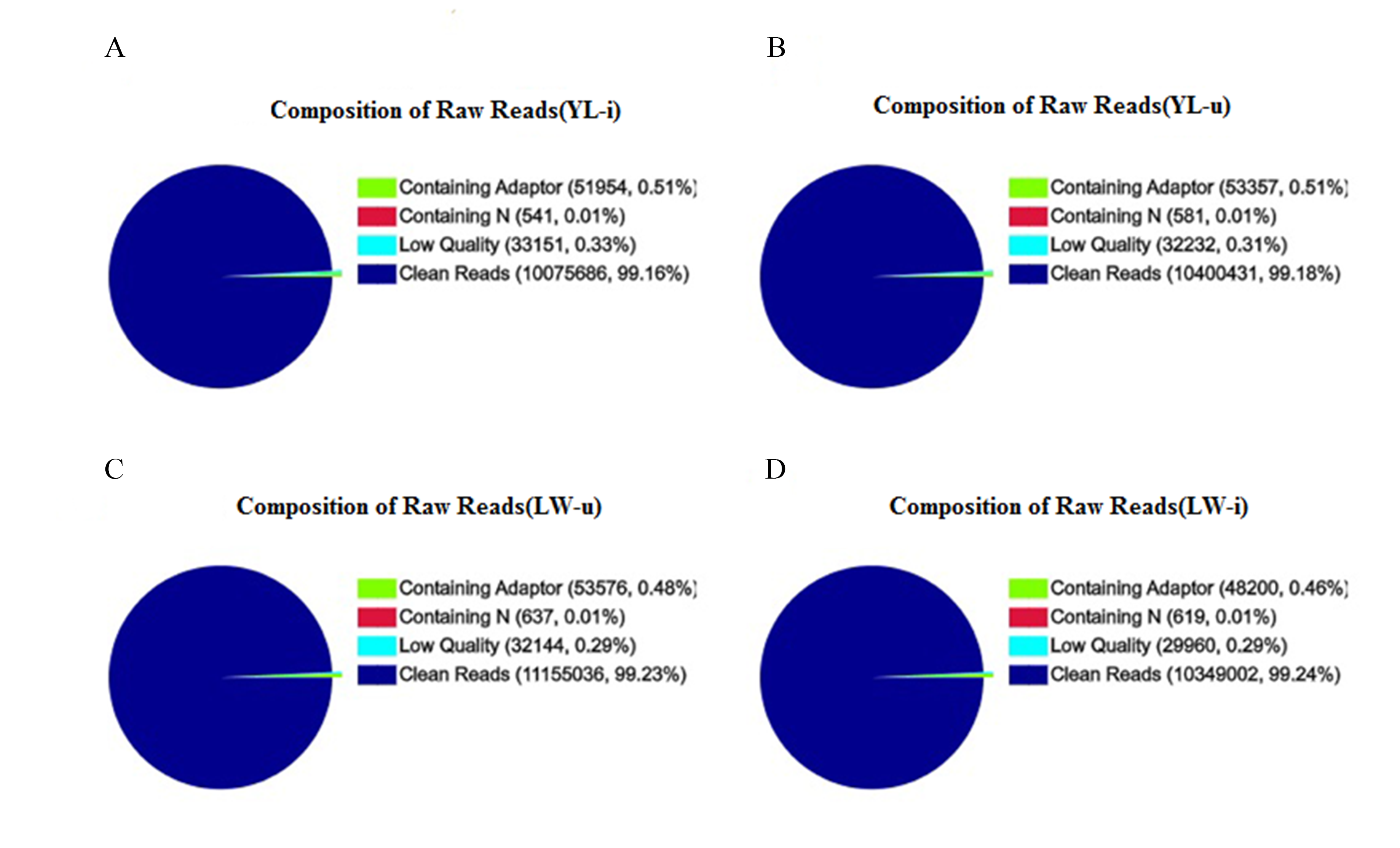

Supplement: S1 Fig — (TIF) [file pone.0155502.s001.tif]

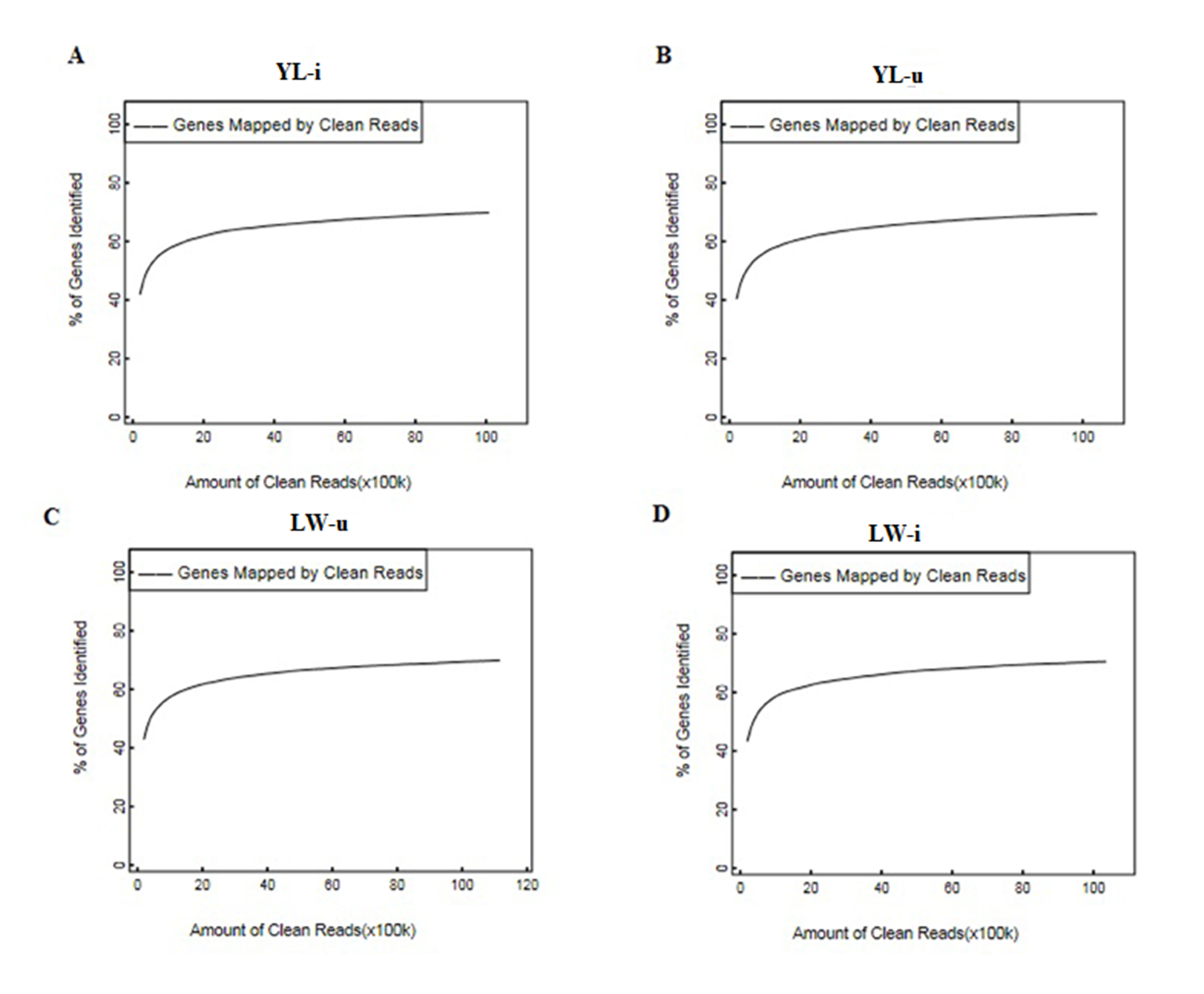

Supplement: S2 Fig — (TIF) [file pone.0155502.s002.tif]

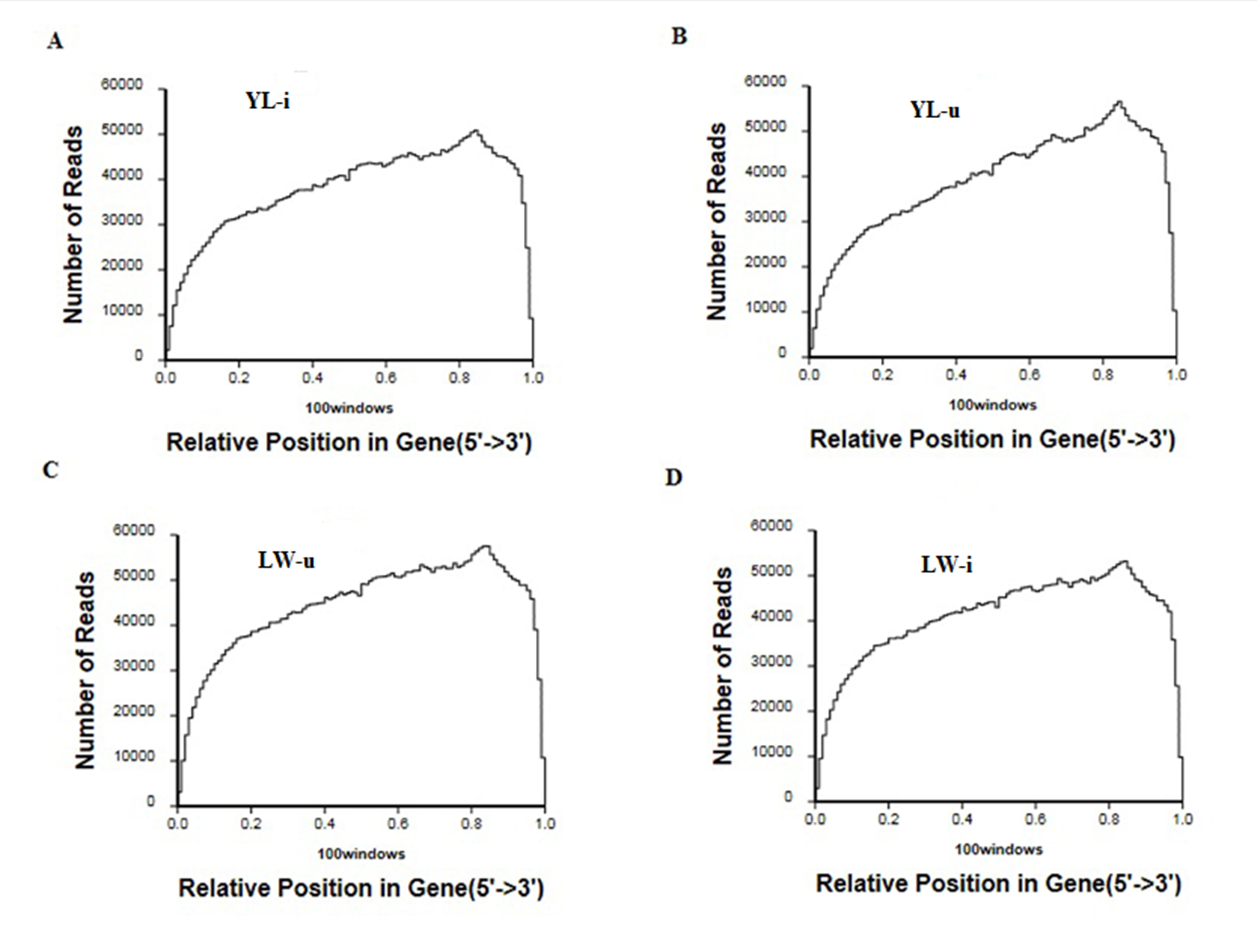

Supplement: S3 Fig — (TIF) [file pone.0155502.s003.tif]

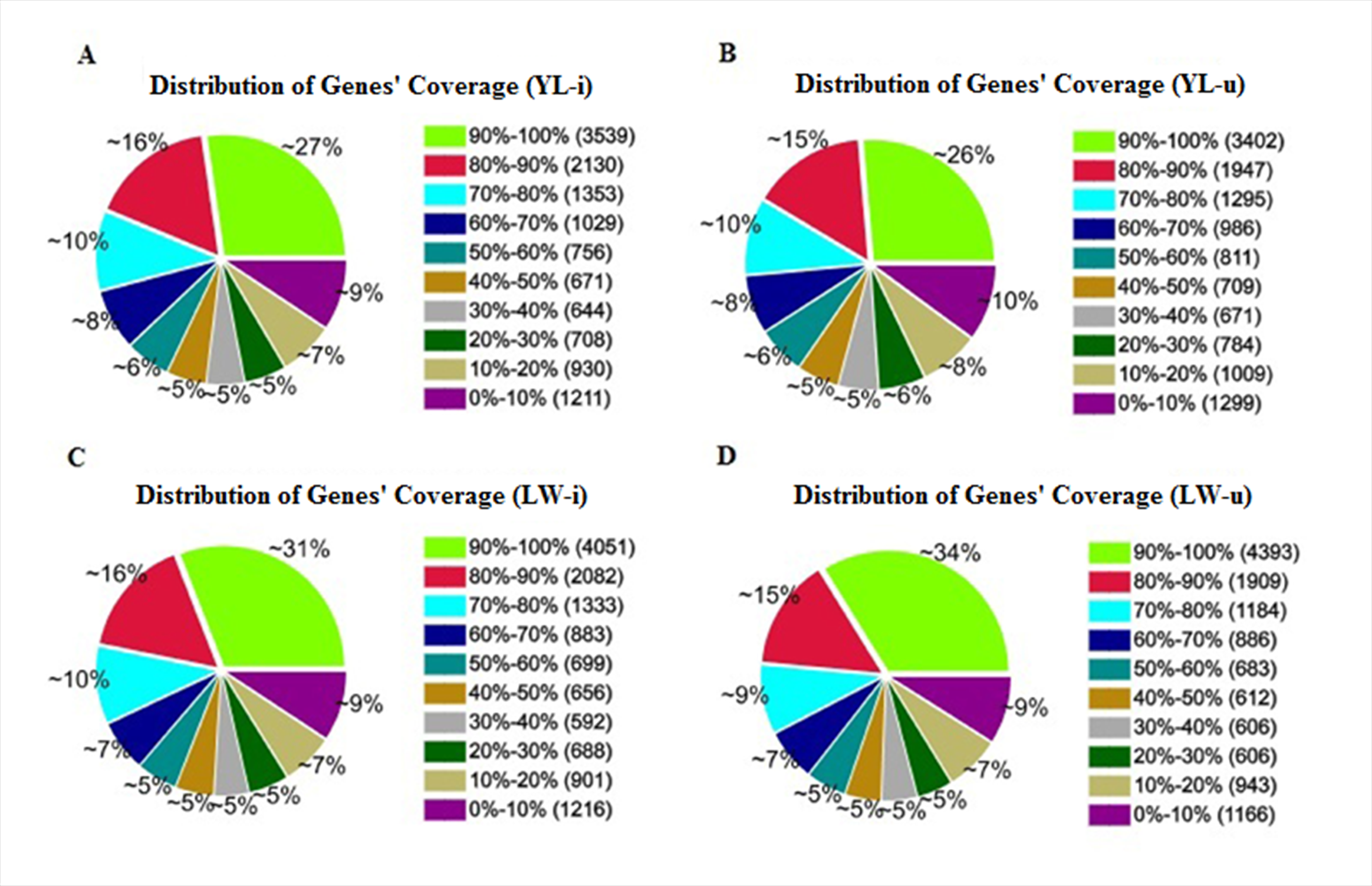

Supplement: S4 Fig — (TIF) [file pone.0155502.s004.tif]

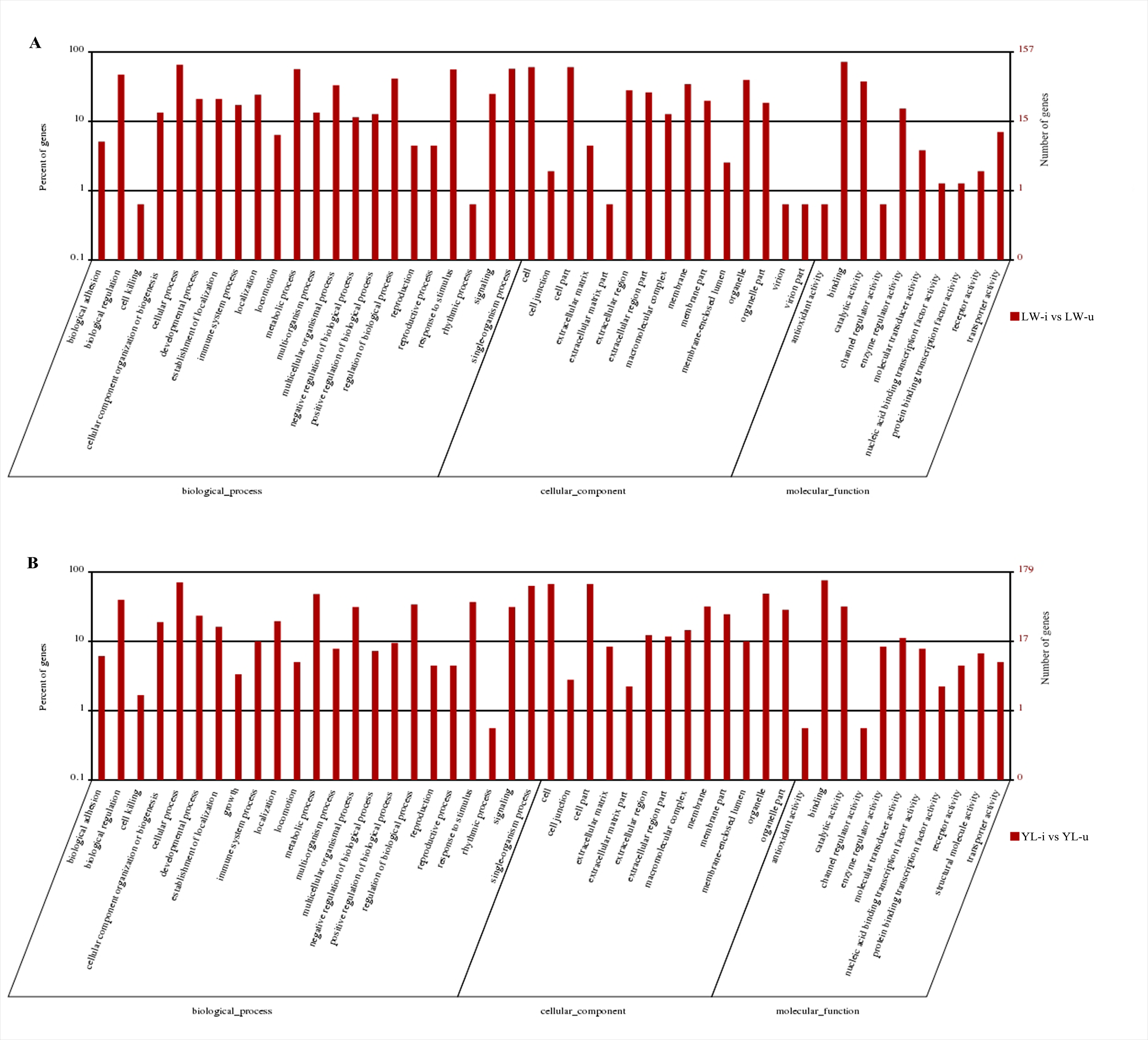

Supplement: S5 Fig — (TIF) [file pone.0155502.s005.tif]
